# The importance of co-pathologies on neuropsychiatric symptoms in dementia

**DOI:** 10.18632/aging.204430

**Published:** 2022-12-09

**Authors:** Lucy L. Gibson, Dag Aarsland, Claudia K. Suemoto

**Affiliations:** 1 Old Age Psychiatry Department, Institute of Psychiatry, Psychology and Neuroscience, King’s College London, UK; 2 Centre for Age-Related Disease, Stavanger University Hospital, Norway; 3 University of São Paulo Medical School, Brazil

**Keywords:** neuropsychiatric symptoms, hallucinations, neuropathology, Alzheimer's disease, Lewy body dementia

Neuropsychiatric symptoms (NPS) such as psychosis, depression, anxiety and apathy are highly prevalent across neurodegenerative dementias and are associated with greater cognitive deterioration, poorer quality of life and increased mortality risk [1]. The profile and evolution of NPS differs across dementia subtypes with clinical studies indicating psychosis is commonest in LBD [2]. There is an urgent need for better treatments of NPS, which requires understanding the underlying pathology. Indeed, NPS often emerge in the prodromal stages of dementia, sometimes termed mild behavioral impairment, suggesting early-stage neuropathology contributes to their etiology [3]. However, most studies rely on post-mortem clinico-pathological correlation in confirmed cases of dementia, usually with end stage disease, and the relationship between neuropathology and NPS in early disease remains unclear. Furthermore, it is increasingly apparent that most patients with DLB have multiple comorbid neuropathologies but the clinical impact of this is unknown [4].

A large population-based cohort, demographically representative of São Paulo, underwent detailed neuropathological phenotyping with clinical data including the neuropsychiatric inventory (NPI) collected from next of kin within 24 hours of death [5]. 1,038 cases were included, classified neuropathologically as meeting criteria for Alzheimer’s Disease (AD *n* = 189); Lewy body disease (LBD *n* = 60); both AD and LBD (AD+LBD *n* = 28) or without sufficient neuropathology to meet criteria for these or other neurodegenerative and cerebrovascular diseases (NP *n* = 761). A wide spectrum of various neuropathologies was seen across all the included cases: neurofibrillary tangles (NFT) Braak stage ≥III (36%), moderate or frequent B-amyloid neuritic plaques (24%), LB Braak stage >III (8%), hyaline atherosclerosis (16.1%), cerebral amyloid angiopathy (CAA) (4%) and TDP-43 (10%) allowing evaluation of low burden of neuropathology to also be assessed.

Cases with dual AD+LBD pathology had the highest relative risk for developing hallucinations, agitation and apathy, and had the greatest total number of symptoms in the NPI. When the neuropathological correlates of NPS were across a range of neuropathological substrates with diagnostic categories collapsed, higher LB Braak stages were associated with increased odds of developing hallucinations (OR [95% CI] = 1.27 [1.11–1.44], *p* < 0.001), independent of cognition and demographic variables. The number of cases with hallucinations increased progressively with higher LB Braak stages. Higher NFT Braak staging was associated with depression, agitation and greater overall burden of NPS. No evidence was found for a multiplicative interaction between LB and AD pathologies. Other neuropathological substrates such as CAA, hyaline atherosclerosis, and TDP-43 did not associate with greater odds of any of the investigated NPS.

This study highlights the additive importance of AD and LBD pathology underpinning NPS, with progression of neuropathology from the early stages increasing the odds of NPS irrespective of the overarching clinicopathological diagnosis. In the population-based sample only 16% met criteria for dementia but NFT and LBD were associated with NPS independent of cognition, emphasizing the contribution of the earliest stages of neuropathology to NPS. NPS are multifaceted, likely with different etiological contributions within and across different dementia subtypes contributing to their inherent clinical heterogeneity. The severity of NPS also appears to directly relate to neuropathology in the later stages of dementia [6]. However, the location of neuropathology is another important factor not fully captured by the Braak staging used in this study. Braak staging reflects the typical progression of LB pathology but does not delineate key nuclei such as the locus coeruleus and is often inadequate where patterns are divergent and pathology is limited to the neocortex or limbic system [7]. This can be addressed in future studies using newer staging classifications and *in vivo* PET imaging.

Co-pathologies in DLB are known to impact clinical phenotype with implications on cognitive deterioration, disease progression and prognosis [4]. Co-pathology also clearly contributes to the clinical variability in NPS and thus is an important consideration for future clinical trials particularly those aiming to develop therapeutic options to treat these heterogeneous symptoms.
